# Quality of life during the COVID-19 pandemic – Results of the CORONA HEALTH App study

**DOI:** 10.25646/8867

**Published:** 2021-10-13

**Authors:** Sophie Eicher, Rüdiger Pryss, Harald Baumeister, Claudia Hövener, Nina Knoll, Caroline Cohrdes

**Affiliations:** 1 Robert Koch Institute, Berlin Department of Epidemiology and Health Monitoring; 2 Julius-Maximilians-Universität Würzburg, Institute for Clinical Epidemiology and Biometry; 3 Universität Ulm, Department of Clinical Psychology and Psychotherapy; 4 Freie Universität Berlin, Department of Education and Psychology

**Keywords:** QUALITY OF LIFE, COVID-19, SOCIO-DEMOGRAPHIC DIFFERENCES, APP-BASED SURVEY

## Abstract

The COVID-19 pandemic has brought about great changes to the everyday lives of the population in Germany. Social distancing, working from home and other measures to contain the pandemic are essentially dominating everyday life. With data from the CORONA HEALTH App study we analysed the quality of life of the adult population in Germany during the COVID-19 pandemic and identified possible risk factors for a poor quality of life. In the app-based survey carried out between July and December 2020, 1,396 respondents (women 46.5%, men 52.7%, diverse 0.9%; mean age (mean) 42.0 years (standard deviation=13.4)) provided information on their quality of life using the World Health Organization Quality of Life Questionnaire (WHOQOL-BREF). Univariate and multivariate regression was used to examine differences in quality of life between different groups of people during the COVID-19 pandemic and their associations with selected predictors. In summary, women, younger persons and job seekers or those who saw their work hours reduced or who could not pursue their regular jobs presented a lower quality of life in individual areas of life than the respective reference group. On the other hand, a setting that combines working from home and at the regular workplace, as well as living together with other people, showed partly positive associations with quality of life. The results have implications for public health interventions as they highlight groups requiring closer attention and sufficient support services.

## 1. Introduction

The COVID-19 pandemic put the health system under great pressure and measures to contain the virus have had impacts both on the economy and society [1–3]. Shops and restaurants either were closed or had their opening hours restricted, school and day-care closures posed major challenges for families, such as having to combine working from home with taking care of children – developments which also potentially exacerbated social inequalities [4]. Many changes and challenges in everyday life are also related to the quality of life of each individual in different domains (i.e. areas of life, Infobox).

The measures to contain the COVID-19 pandemic have resulted in fundamental changes to everyday life for many people. Experiencing negative emotions, such as fear of infection, concern about the health of family members and stress, for example due to the dual burden of working from home and childcare, are among the negative consequences being discussed [8]. For many people, uncertainties regarding their physical well-being or income security have also played a major role during the pandemic [9, 10], as well as dealing with the mandatory social distancing measures [9, 11]. Scientific findings to date suggest that the COVID-19 pandemic has impacted various domains of quality of life for some population groups more markedly than for others. For example, international studies have found an increased risk for a decrease of quality of life during the COVID-19 pandemic for women, jobseekers and younger people [12–14]. Individuals with higher educational status generally appear to score higher on quality of life during the pandemic than individuals with lower educational status [12, 14, 15]. On the other hand, in China, individuals with a higher educational status appear to suffer higher levels of psychological strain [16]. Health care sector employees seem to be particularly challenged within the current circumstances. This is supported by findings of an increase in depression symptoms and anxiety as well as feelings of acute stress during the COVID-19 pandemic among health care sector employees in China and India [17–19]. Parents faced with frequently having to combine home schooling and working from home also report being stressed by their situation, and specifically in Germany single parents and mothers are apparently most affected [8, 20]. Some evidence also indicates potential factors that have enhanced quality of life during the COVID-19 pandemic. Household size and living conditions have for example been associated with quality of life. Since social distancing measures have severely limited contact outside one’s own household, living in a larger household could protect against social isolation and its potentially negative effects on satisfaction in life [21]. Both before and during the COVID-19 pandemic, being able to access a balcony, terrace or garden has been positively associated with quality of life in Germany [22, 23]. In summary, international studies so far conclude that certain groups of people may be at an increased risk of poorer quality of life during the COVID-19 pandemic.

In contrast to findings from before the COVID-19 pandemic, the quality of life of younger rather than older people has proved to be poorer. Findings regarding a poorer quality of life for women compared to men and jobseekers compared to people who have a job appear to have remained unchanged. The extent to which the COVID-19 pandemic amplifies differences in subjective quality of life for certain groups of people is not yet clear and will require longitudinal studies. The CORONA HEALTH App study aims to identify factors indicating possible risks of a reduction in quality of life during the COVID-19 pandemic in Germany. Therefore, (a) the quality of life of the adult population in Germany during the COVID-19 pandemic will be investigated and (b) possible risk factors of a reduced quality of life identified. To this end, both a general assessment of subjective quality of life (overall quality of life) and of its four domains (psychological, physical, social, environment-related) will be made.

**Info box quality of life**

This paper uses the term quality of life in the sense of the World Health Organization (WHO) definition, which also guides the questionnaire used in the study. The WHO defines quality of life as ‘an individual’s perception of their position in life in the context of the culture and value systems in which they live and in relation to their goals, expectations, standards and concerns’ [5]. The WHO categorises quality of life based on four domains: psychological, physical, social relationships and environment. Psychological quality of life ‘represents the general mental state’ of a person [6]. This includes the extent to which a person experiences positive feelings or is burdened by negative ones [6]. Physical quality of life ‘maps a person’s overall physical condition’ [6]. This covers the limitations people experience in everyday life for example due to pain, physical complaints or sleep problems [6]. The social relationships quality of life domain reflects how satisfied a person is, for example, with their social relationships and with the social support they receive from friends [6]. The environment-related quality of life domain covers how secure a person feels in their daily life. This covers, among other aspects, satisfaction with their current financial situation, access to information and also access to health services [6]. As an indicator of subjective well-being, quality of life is of particular public health relevance [7].

## 2. Methodology

### 2.1 Sample design and study implementation

Data for this survey was collected via the CORONA HEALTH App. The app was developed as a result of a joint project between the Robert Koch Institute and the Julius Maximilian University of Würzburg, as well as the universities of Ulm and Regensburg. Data collection for the CORONA HEALTH App study on adult mental health began in July 2020 and is ongoing. After taking part in the twenty-minute baseline survey, respondents can opt to take part in a follow-up survey of about ten minutes. Respondents are required to be at least 18 years old [24]. This paper only considers data collected during the cross-sectional baseline survey between 23 July 2020 and 1 December 2020. This period was comprised of a phase in which the first infection wave was subsiding with few pandemic-related containment measures (e.g. compulsory testing for travellers returning home, social distancing and hygiene rules) as well as a period of rising incidence (the second COVID-19 wave) with a corresponding tightening of measures (‘lockdown light’; e.g. tightening of social distancing recommendations) [25]. The sample comprised a total of 1,396 persons (women=46.5%, men=52.7%, diverse=0.9%) aged 18- to 84-years (mean (M)=42.0; standard deviation (SD)=13.4). For the planned analyses, the desirable sample size was set to at least 236 persons in order to achieve a sufficiently high test strength of 0.9. The calculation was carried out with G*Power for a multiple regression model with fixed effects (R^2^ deviation from zero) with the following values: effect size f^2^ =0.1; alpha error probability =0.05; power =0.9; number of predictors =13. Due to the low number of cases for inferential statistical testing of group differences, individuals with diverse genders were not included in regression analyses.

### 2.2 Study variables

As part of the baseline survey, participants first were required to answer a number of questions on socio-demographic factors, followed by questions on their mental health and quality of life. For this paper, the following information was used: age, number of household members, gender, education, work status (i.e. whether the person has reduced working hours), their place of work (i.e. whether the person works from home), health care profession (i.e. whether the person works in a health care profession), housing situation (i.e. whether the person has access to a garden/balcony/terrace), number of children in the household, childcare and working from home, lifetime diagnosis of a mental disorder, presence of a chronic illness or a COVID-19 infection (Table 1).

In addition, the World Health Organization Quality of Life Questionnaire (WHOQOL-BREF) [26] was used as an indicator of quality of life in the four domains (1) psychological quality of life (e.g. ‘Do you consider your life meaningful?’); (2) physical quality of life (e.g. ‘How satisfied are you with your sleep?’); (3) social quality of life (e.g. ‘How satisfied are you with your social support?’) and (4) environment-related quality of life (e.g. ‘How satisfied are you with your living conditions?’). The participants answered each item on a five-point scale ranging from 1= ‘Not at all’ to 5= ‘Completely’ or from 1= ‘Very dissatisfied’ to 5= ‘Very satisfied’. Furthermore, overall quality of life was evaluated separately (‘How would you rate your quality of life?’; 1= ‘Very poor’ to 5= ‘Very good’) [26]. The reliabilities of the individual subscales range between α=0.74 for the social quality of life domain and α=0.91 for the physical quality of life domain [6]. The evaluation of the individual domains of quality of life was carried out according to the WHOQOL-BREF manual via scale transformation of the sum values to a scale from 0 to 100 [26], higher values indicate a better quality of life.

Education was categorised as low, medium and high according to the international classification Comparative Analyses of Social Mobility in Industrial Nations (CASMIN) [27] (low = no school-leaving certificate/secondary school leaving certificate, medium = secondary school leaving certificate/polytechnical secondary school, high = vocational baccalaureate/baccalaureate). In addition, due to systematically missing values, the variable ‘place of work’ was recoded for the analysis. People who were not in regular employment at the time of the survey because they were already retired for example, did not respond to the item and were assigned to the newly formed category of ‘currently not in regular work’. Consequently, four categories emerged for the variable ‘place of work’: working at the regular place of work, working both from home and at the regular place of work, working from home and currently not in regular employment. For the variable work status, the categories ‘reduced working hours due to COVID-19’, ‘No regular work due to closed day-care centres or schools’ and ‘No regular work due to health protection measures’ were combined into the category ‘No regular work due to COVID-19 measures’. As the groups of pensioners and housewives were not the focus and did not differ systematically in connection with quality of life, they were combined for reasons of clarity and reduction of complexity. More detailed information on the sample and the distribution of the variables used can be found in Table 1. There were no missing values.

### 2.3 Statistical evaluation

In this article, we aim to monitor dynamics of quality of life during the COVID-19 pandemic. A robust univariate regression was calculated with the overall quality of life as criterion and the socio-demographic (e.g. age, sex) and relevant control variables (e.g. chronic disease) and, secondly, a robust multivariate regression with the four quality of life domains as criteria and with the same predictors and control variables.

In addition, to obtain findings on differences in quality of life over the course of the COVID-19 pandemic, the sample was divided into two temporally separate groups: the first period covers mid-July to mid-September, the second mid-September to early December. While in the first period infection rates were low and relatively stable, the second period is characterised by an increasing number of SARS-CoV-2 infections and the tightening of measures (e.g. ‘lockdown light’ beginning in November). Linear regression was used to test for differences between the two periods. The significance level was 5% so a statistically significant difference was assumed for p-values <0.05.

The analyses were calculated with RStudio version 1.3.1093 [28] Initially, the data from 1,406 respondents were checked for inconclusive answering patterns by means of various indicators (straightlining index, longstring index, intra-individual response variability and Mahalanobis Distance) with the R package careless (v1.1.3, [29]) and plausibility checks were carried out for selected answers (e.g. consistency of answers to similar or contrary questions). Based on the test results, the response of ten participants was deemed unreliable and these respondents subsequently excluded.

## 3. Results

**Figure 1:**
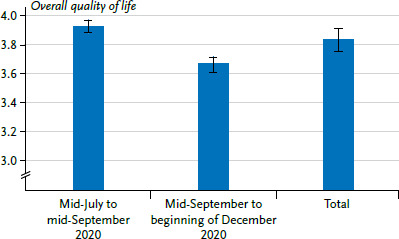
Means and confidence intervals for overall quality of life (scale 1 to 5) for the period covering the months from mid-July to mid-September 2020 (n=933), mid-September to early December 2020 (n=463) and overall (n=1,396). For exact values see Table 2 Source: CORONA HEALTH App study

**Figure 2:**
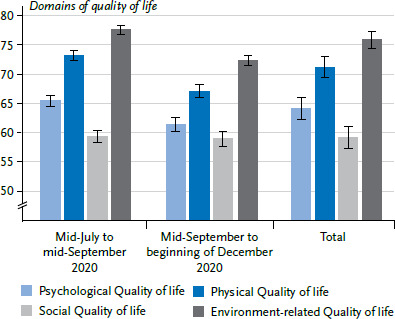
Means and confidence intervals in the four domains of quality of life (sum scores 0 to 100) for the period covering the months from mid-July to mid-September 2020 (n=933), mid-September to early December 2020 (n=463) and overall (n=1,396). For exact values see Table 2 Source: CORONA HEALTH App study

A final sample of 1,396 persons was included in the analyses (n=649 women (46.5%), n=735 men (52.7%), n=12 divers (0.9%)). The mean age of respondents was 42.0 years (SD=13.4, min=18, max=84). At the point of the survey, ten people had an acute COVID-19 infection (0.7%), while 1,386 reported that they did not have COVID-19 or had already recovered (99.3%). People with a high educational status are overrepresented (72.0%), whereas older adults are underrepresented. Also, a relatively large number of people (36.3%) self-reported a diagnosis of a mental disorder (based on diagnostic interview, some 27.7% of adults living in Germany suffer some kind of mental disorder [30]). The reported results refer to the period from 23 July 2020 to 1 December 2020. For the item to assess the overall quality of life, a mean value of 3.84 (SD=0.88) on a scale of 1 to 5 was obtained. The figures and table 2 show the respective mean values and confidence intervals for the entire item (Figure 1, Table 2) and the domains (Figure 2, Table 2) over time. Higher values indicate a higher quality of life.

### 3.1 Overall quality of life

The univariate regression model showed a correlation of global quality of life with age and educational status but not with the sex of the study participants (Table 3). Older individuals and those with higher education reported a better overall quality of life than younger individuals and those with lower education. Findings for overall quality of life also differed depending on participant’s current work status: individuals, who are not in regular employment due to the COVID-19 restrictions, people on sick leave due to a condition other than COVID-19, pensioners, as well as the group of housewives or househusbands, reported a lower global quality of life than individuals with regular work (Table 3). The overall quality of life values for jobseekers at the time of the survey did not differ significantly from people in regular employment (Table 3). For those in regular employment, there was a correlation with overall quality of life in terms of place of work: people who worked both from home and at their regular workplace had a higher global quality of life compared to people who worked mainly at their regular workplace (Table 3). However, no differences were found for people who worked at their regular workplace compared to those working from home or people without a regular job (Table 3). No correlations with overall quality of life were found for people working in the health care sector or regarding housing conditions (Table 3). There were also no differences in overall quality of life depending on household size and number of children. Overall quality of life for parents working from home also did not differ from the values found for other participants.

### 3.2 Psychological quality of life

The multivariate regression model showed a correlation between quality of life in terms of mental health with age, sex and educational status (Table 3). Older respondents, men and those with higher education reported better mental health than younger respondents, women and those with lower education. Respondents who were not in regular work at the time of the survey due to COVID-19 restrictions, were on sick leave due to a condition other than COVID-19, or who were seeking work reported poorer mental health than individuals in regular employment (Table 3). A correlation between mental health and place of work is found for those in regular employment: better mental health is found in people who work both from home and at their regular workplace compared to people who work mainly at their regular workplace (Table 3). No other correlations between the variables studied and psychological quality of life were found (Table 3).

### 3.3 Physical quality of life

For physical quality of life, the multivariate regression model highlighted a correlation between age, sex and educational status (Table 3). Older respondents, men and those with higher education reported a higher physical quality of life than younger respondents, women and those with lower education (Table 3). Further correlations with physical quality of life were found for work status (Table 3). Persons in receipt of pensions, housewives and househusbands, persons who were not in regular work at the time of the interview due to COVID-19 measures, were on sick leave due to a condition other than COVID-19, or were seeking work reported lower levels of physical quality of life than persons with regular working hours (Table 3). No other correlations between the variables studied and physical quality of life were found (Table 3).

### 3.4 Social quality of life

For social quality of life, the multivariate regression model showed a correlation between age, gender and educational status (Table 3). Older respondents, women and those with higher education reported a higher social quality of life than younger respondents, men and those with lower education (Table 3). No correlations with social quality of life were found for work status and place of work (Table 3). Respondents who work in the health sector reported a higher average social quality of life than people from other occupational groups (Table 3). Housing conditions were not related to social quality of life, but a positive relationship with household size was found. Respondents with children, however, reported a lower social quality of life compared to those without children. No other correlations between the variables studied and social quality of life were found (Table 3).

### 3.5 Environment-related quality of life

The multivariate regression model revealed a correlation between environment-related quality of life with age, educational status, but not with sex (Table 3). Older respondents and those with higher education reported a higher environment-related quality of life than younger respondents and those with lower education. A negative association with environment-related quality of life was found for people without a regular job due to the COVID-19 restrictions and for people who were on sick leave due to a condition other than COVID-19 (Table 3). People who had a balcony, garden or terrace reported on average a higher environment-related quality of life than people without a balcony, garden or terrace. No other correlations between the variables studied and environment-related quality of life were found (Table 3).

### 3.6 Control variables

Respondents suffering from mental health issues and chronic illnesses reported a lower quality of life in all domains than those without pre-existing conditions (Table 3). Individuals without an acute COVID-19 infection at the time of the interview did not differ from other respondents in terms of psychological, physical, social and environment-related quality of life. However, they had a significantly better overall quality of life.

### 3.7 Differences by time period covered

With the exception of social quality of life, differences in the quality of life of study participants were found after controlling for age, sex, education, chronic illness or self-reported lifetime diagnosis of a mental health issue, depending on the period of time covered. Overall quality of life (B=-0.26, SE=0.05, p<0.001), as well as psychological quality of life (B=-4.00, SE=1.15, p<0.001), physical quality of life (B=-6.11, SE=1.08, p<0.001) and environment-related quality of life (B=-5.38, SE=0.87, p<0.001) were lower for study participants in the later time period (social quality of life: B=-0.36, SE=1.25, p=0.77).

## 4. Discussion

The present study analysed the quality of life of adults living in Germany during the COVID-19 pandemic for the period stretching from 23 July 2020 to 1 December 2020 and aimed to identify groups that could potentially be affected by a poor quality of life.

A key finding is that the quality of life of older people is better than that of younger people. Other studies also conclude that older people do not have a higher risk of a poorer quality of life during the pandemic [31, 32]. It should be noted that the average age of the sample was 42.0 years in the present study. Significantly fewer people from the 63+ age group participated in the survey. One possible reason for this is that the survey was conducted via an app and that older people and pensioners in particular are less likely to have a smartphone than younger people (87% to 97% of people aged 20 to 60 owned a smartphone in 2019 and 43% to 73% of people aged 60+) [33]. Furthermore, the extent to which older persons in nursing homes or homes for the elderly are represented in this study is unclear. Due to stricter social distancing measures, people in these settings may face a greater risk of a poor social quality of life [34]. In contrast to previous findings [35], and based on these study data, older age cannot generally be considered a risk factor for poorer quality of life during the COVID-19 pandemic. Older people may even be more capable of mobilising resources to maintain their quality of life [32]. Based on these results, younger age can be assessed as representing a risk factor for poorer quality of life during the COVID-19 pandemic. Possible reasons are uncertain education, training and employment conditions at an age in any case characterised by change [12, 13]. Potentially, younger people also experience the restrictions imposed to control infections (e.g. studying and working from home) as more limiting compared to older people [12, 13].

In line with previous findings on quality of life in general, but also during the COVID-19 pandemic [12, 35, 36] differences in the perception of quality of life were found between women and men. Men reported higher levels of psychological and physical quality of life than women, but lower levels of social quality of life. Although the results of studies on women’s social well-being during the COVID-19 pandemic are inconsistent, some findings indicate that women could be more active in terms of maintaining social contacts (e.g. by phone [37]) as well as better with social coping strategies (e.g. seeking social support [38]). These findings could hold some explanatory significance, however, they will require corroboration through further evidence. Regardless of the current pandemic, women report an on average poorer quality of life than men, [35, 39] and thus generally have an increased risk of lower psychological and physical quality of life.

Another key finding of this study is that people with higher educational status had a higher quality of life across all domains compared to people with lower educational status. This result is consistent with the findings of other studies [12, 14]. These findings were also found in studies regardless of the pandemic [40]. As such, the association between educational status and quality of life during the pandemic was to be expected.

As in other studies, jobseekers at the time of the survey reported lower levels of psychological and physical quality of life [12, 41]. The analyses show that being employed during the pandemic was a protective factor for quality of life [41]. Regardless of the pandemic, frequently replicated findings show that unemployment in general negatively impacts well-being and life satisfaction [42–44]. The present study supports the current state of research, which indicates that this correlation persists during the COVID-19 pandemic and especially impacts the domains of psychological and physical quality of life.

For people who due to the pandemic do not work their regular hours, for example because they have reduced working hours, there is a negative correlation with the overall quality of life and specifically with the following domains of quality of life: psychological, physical and environment-related. To date, we know of no studies that deal specifically with the quality of life of this group of people. One possible interpretation for the persons affected could be financial difficulties and fears regarding job security. However, further studies are needed here to analyse the correlations between changed working conditions due to COVID-19 related restrictions and quality of life.

Contrary to other studies [17], the study found no indication of a poor quality of life for health care professionals. When interpreting the results, it must be noted that the CORONA HEALTH App study made no distinction between different health care professions. As has been shown [18], levels of anxiety are greater among nurses than among doctors for example, which highlights the need to differentiate between health care professions. Presumably, other factors also play a role for the quality of life of people working in these professions. For example, health literacy reduces the figures for depression symptoms [45]. Here health literacy refers to the ability to access and understand health information and to make adequate decisions on the basis of this information [46]. Health care professionals are presumably better informed about the virus than other people, which could mean that due to health literacy their quality of life is less affected. Furthermore, this group of people reported a higher social quality of life than other people. A possible explanation could be that they spend more time with colleagues, i.e. they continue to work in their regular work environment, so that, in spite of social distancing measures, they have more contact with other people. Possibly, due to the important role they play in the pandemic, people in this group also receive more recognition from their social environment. Further studies will have to clarify these post hoc assumptions.

Contrary to what was expected, no evidence was found for a reduction in quality of life for parents working from home compared to other people (i.e. parents who do not work from home and persons without children). The results could not confirm the assumption that the double-burden of having to take care of children while working from home leads to more stress for parents and negatively impacts quality of life. However, using the data available, it is not possible to distinguish between parents who live in a household with their children and work from home and parents who do not live in a household with their children and work from home. In a survey by the KKH Kaufmännische Krankenkasse [47] around half of all mothers (49%) and fathers (42%) stated experiencing stress due to childcare. It is possible that people who are stressed do not want to additionally participate in a study and that the sample is therefore biased. Switching to working from home due to the COVID-19 pandemic has also provided some people with more time to relax, for example by eliminating commuting [48]. Thus, it is possible that for some parents, working from home during the pandemic may provide relief. Furthermore, flexible working hours or other variables that have not been considered could play a role in these findings. Physical activity, for example, has a moderating influence on the relationship between stress perception and quality of life of mothers working from home [15]. Based on the present study, parents working from home therefore do not generally see their risk of a lower quality of life increased, but a more detailed analysis of other factors, such as stress or physical activity, would have to be taken into account.

A positive correlation between household size and social quality of life was also evident. Social proximity to other people, i.e. living with other household members could have a positive effect, offsetting the effects of social distancing measures that were implemented as protective measures to contain the COVID-19 pandemic. Further studies have also identified living with other adults as a factor protecting well-being during the COVID-19 pandemic [49].

People with children reported a poorer social quality of life than people without children. This result is consistent with general findings on the quality of life of parents [50] and thus indicates that during the pandemic parents also have an increased risk of suffering a lower social quality of life. For parents with small children of day-care and primary school age, general satisfaction as well as satisfaction with family life also decreased compared to before the pandemic [51, 52]. Mothers in particular reported dissatisfaction with family life, whereas fathers reported greater satisfaction [52].

Consistent with other studies [22, 23] the results suggest that the environment-related quality of life of people with access to a balcony, terrace or garden is better than that of people without such access. For example, garden owners reported greater well-being compared to people without a garden [22, 23]. Not being able to access a balcony or garden directly correlated to a lower quality of life [23]. The present study thus supports the assumption regarding an association between housing conditions and environment-related quality of life, which, however, may also exist irrespective of the COVID-19 pandemic. Overall, the reported quality of life scores, with the exception of social quality of life, were above the global norm scores for the WHOQOL-BREF questionnaire [53]. This can be interpreted as indicating a relatively high quality of life in international comparison. This is also supported by the fact that the quality of life in the study sample is consistently higher than in an Italian sample with a comparable period of data collection during the COVID-19 pandemic [12].

There are no current population-representative norm values for the German WHOQOL-BREF; it was last used in Germany by a representative study in 2004 [54] and in a short form in 2007 [55]. This makes it difficult to ensure a reliable discussion of possible differences in quality of life compared to the period before the pandemic. The data from 2004 and 2007 can only be used as a rough guide, as well as a recent publication that also examines quality of life using WHOQOL-BREF during the COVID-19 pandemic [56]. With the exception of environment-related quality of life, the values in the present study are significantly lower than in 2004 (e.g. mean value of psychological quality of life in 2020 =64.17 and in 2004 =73.13 [54]). The global quality of life, on the other hand, with a value of 3.84 in 2020, corresponds exactly to the 2007 mean value [55]. The extent to which these differences are related to the COVID-19 pandemic cannot be reliably assessed, however, a fact which calls for longitudinal observations of quality of life over the course of the COVID-19 pandemic. To date, the longitudinal findings in the present study suggest that quality of life does change in relation to the dynamics of COVID-19 infections. Between mid-September and early December 2020, a period marked by rising COVID-19 infection figures and stricter non-pharmaceutical measures (e.g. wearing face masks), study participants reported poorer quality of life than during the relatively stable summer months (mid-July to mid-September 2020). However, it is reported that quality of life scores were lowest in May and then stabilised from July onwards [57]. The mean values presented for the individual domains are lower than in another study that examined quality of life during the COVID-19 pandemic. However, this study only reports data from May 2020 whereas the results presented here also cover later periods when the pandemic was more advanced [56].

In summary, based on the data collected, women, younger persons and job seekers or those who saw their working hours affected due to the COVID-19 restrictions are the groups facing an increased risk of a lower quality of life in individual domains. These groups should therefore be given increased attention and support services made available to them during the COVID-19 pandemic. Furthermore, the results indicate that parents are at increased risk of lower social quality of life, even though this does not seem to be directly related to the work situation at home (working from home). Further studies should focus on taking a more differentiated look at mechanisms that could explain this fact, for example by analysing stress experience and stressors.

### Limitations

It should be noted that the reported results are subject to some limitations. As a convenience sample, the sample is not representative of the adult population living in Germany. In particular, respondents with a high educational status are overrepresented, older adults in turn are under-represented and the proportion of participants with a self-reported diagnosis of a mental health issue is relatively high. Therefore, the present study cannot make generalised statements about all population groups living in Germany, but indications of significant correlations and risks can be derived, which should be taken up and considered in more detail by further research. Furthermore, additional factors that were not considered by the present study could have influenced the presumed correlations. Therefore, future studies will need to clarify the role of the possible moderating and mediating variables that were identified (e.g. health literacy).

It should also be noted that the data refers to the period from 23 July 2020 to 1 December 2020. This included a low incidence period (the summer of 2020), but also covers part of the second COVID-19 wave [25]. Further studies of quality of life are needed, especially to consider the subsequent pandemic stages. To identify possible differences or long-term and short-term changes in the quality of life over time and in relation to infection levels, further analyses and longitudinal data are also necessary.

Furthermore, there are only a few studies undertaken in Germany to date that deal with the correlations examined. Therefore, studies from other countries and cultural environments, which may have reacted differently to the pandemic and the implementation of restrictions, were used. The present study results cannot be generalised, but they can provide valuable insights for follow-up studies aiming to analyse quality of life during the COVID-19 pandemic in Germany.

## Key statements

- Women and younger people have an increased risk of a poorer quality of life in certain areas of life.
- Jobseekers and people whose regular jobs have been constrained by the measures to contain the COVID-19 pandemic are at a higher risk of poorer quality of life in certain areas of life.
- People who can combine work at their regular workplace with working from home have a better overall quality of life.
- People with access to a balcony, garden or terrace have a higher environment-related quality of life.
- The number of household members is positively related to social quality of life, but this does not apply to the number of children.

## Figures and Tables

**Table 1:** Description of the sample of 1,396 participants in the CORONA HEALTH App study for the period covering the months from July to December 2020 Source: CORONA HEALTH App Study

Variable/Groups	n	%
**Gender**		
Women	649	46.5
Men	735	52.7
Diverse	12	0.9
**Education status (CASMIN)**		
Low	82	5.9
Medium	309	22.1
High	1,006	72.0
**Employment status**		
Regular working hours	950	68.0
No regular work activity due to COVID-19 measures	146	10.5
Quarantine (infected with COVID-19 or suspected case)		0.9
Sick leave (illness other than COVID-19)	44	3.1
Jobseeker	61	4.4
Housewife/-husband or pensioner	183	13.1
**Place of work**		
Regular	531	38.0
Working from home	384	27.5
Partly working from home, partly at the regular place of work	216	15.5
Not working regularly	266	19.0
**Health care professional**		
Yes	223	16.0
No	1,174	84.0
**Housing situation**		
Access to balcony/garden/terrace	1,091	78.1
No access to balcony/garden/terrace	306	22.0
**Children**		
Yes	639	45.7
No	758	54.3
**Lifetime diagnosis of mental illness**		
Yes	507	36.3
No	890	63.7
**Chronic disease**		
Yes	606	43.4
No	791	56.6
**COVID-19 status**		
Acute COVID-19 infection	10	0.7
Not currently suffering from/recovering from COVID-19	1,387	99,3

CASMIN=Comparative Analyses of Social Mobility in Industrial Nations, COVID-19=Coronavirus Disease 2019

**Table 2:** Means and confidence intervals for overall quality of life (scale 1 to 5) and the four domains of quality of life (sum scores 0 to 100) over time Source: CORONA HEALTH App study

	July to mid-September 2020		Mid-September to beginning of December 2020		Total	
	M	(CI)	M	(CI)	M	(CI)
Overall quality of life	3.93	(3.87–3.98)	3.67	(3.58–3.75)	3.84	(3.79–3.89)
Psychological quality of life	65.5	(64.2–66.8)	61.5	(59.6–63.4)	64.2	(63.1–65.2)
Physical quality of life	73.2	(72.1–74.4)	67.1	(65.2–69.0)	71.2	(70.2–72.2)
Social quality of life	59.4	(58.0–60.8)	59.0	(57.0–61.0)	59.2	(58.1–60.4)
Environment-related quality of life	77.6	(76.7–78.6)	72.3	(70.7–73.8)	75.9	(75.1–76.7)

M=mean, CI=confidence interval

**Table 3:** Results of robust univariate (overall quality of life) and multivariate (psychological, physical, social and environment-related quality of life) regression analysis (n=1,384) Source: CORONA HEALTH App study

	Overall quality of life				Psychological quality of life				Physical quality of life			
Corrected R^2^	0.18				0.27				0.32			
	B	SE	β	p	B	SE	β	p	B	SE	β	p
Intercept	2.49	0.36	0	**<0.001**	52.96	8.07	0	**<0.001**	55.90	7.29	0	<0.001
Age	0.01	0.34	0.13	**<0.001**	0.30	0.05	0.21	**<0.001**	0.18	0.05	0.13	<0.001
**Gender**												
Women	Reference category											
Men	0.03	0.05	0.02	0.530	2.78	0.97	0.06	**0.004**	2.71	0.92	0.06	**0.003**
**Education status (CASMIN)**												
Low	Reference category											
Medium	0.35	0.11	0.17	**0.001**	6.47	2.37	0.17	**0.006**	7.36	2.21	0.20	**0.009**
High	0.47	0.10	0.24	**<0.001**	8.03	2.24	0.18	**<0.001**	9.09	2.10	0.20	**<0.001**
**Employment status**												
Regular working hours	Reference category											
No regular work activity due to COVID-19 restrictions	-0.19	0.07	-0.07	**0.009**	-4.96	1.57	-0.08	**0.002**	-6.08	1.45	-0.10	**<0.001**
Quarantine (infected with COVID 19 or suspected case)	0.33	0.25	0.04	0.190	-0.96	6.90	-0.00	0.889	-1.76	5.70	-0.01	0.758
Sick leave (illness other than COVID-19)	-0.52	0.16	-0.10	**0.001**	-9.60	3.41	-0.11	**0.005**	-19.20	2.95	-0.21	**<0.001**
Jobseeker	-0.24	0.14	-0.06	0.081	-6.82	2.91	-0.07	**0.019**	-5.91	2.70	-0.06	**0.029**
Housewife/-husband/Pensioner	-0.26	0.12	-0.10	**0.028**	-1.99	2.51	-0.03	0.427	-6.42	2.55	-0.11	**0.012**
**Place of work**												
Regular	Reference category											
Working from home	0.01	0.05	0.01	0.805	-1.01	1.24	-0.02	0.414	-1.03	1.11	-0.02	0.355
Combination working from home/regular	0.14	0.06	0.06	**0.026**	3.50	1.32	0.08	**0.008**	0.26	1.24	0.01	0.836
Not working regularly	0.01	0.11	0.00	0.931	-1.53	2.28	-0.03	0.502	-1.17	2.22	-0.02	0.598
**Profession in the health sector**												
No	Reference category											
Yes	0.06	0.06	0.03	0.291	1.68	1.28	0.03	0.188	1.19	1.20	0.02	0.322
**Housing situation**												
Access to balcony/garden/terrace	Reference category											
No access to balcony/garden/terrace	-0.09	0.06	-0.04	0.106	-0.08	1.22	-0.00	0.947	1.08	1.14	0.02	0.345
Number of household members	0.03	0.02	-0.04	0.169	-0.07	0.43	-0.01	0.867	0.28	0.38	0.02	0.465
**Children**												
No	Reference category											
Yes	0.00	0.06	0.00	0.970	1.01	1.29	0.02	0.433	-0.09	1.23	-0.00	0.941
**Combination childcare/home office**												
No	Reference category											
Yes	-0.06	-0.02	0.10	0.503	-3.74	2.15	-0.06	0.082	-1.32	1.98	-0.02	0.504
**Control variables**												
**Lifetime diagnosis of mental illness**												
No	Reference category											
Yes	-0.42	0.05	-0.23	**<0.001**	-14.41	1.10	-0.36	**<0.001**	-10.95	1.01	-0.27	**<0.001**
**Chronic disease**												
No	Reference category											
Yes	-0.42	0.04	-0.13	**<0.001**	-4.96	1.01	-0.11	**<0.001**	-10.61	0.94	-0.24	**<0.001**
**COVID-19-status**												
Currently suffering from COVID-19	Reference category											
Currently not ill with COVID-19/recovered	0.87	0.32	0.08	**0.007**	1.92	7.49	0.01	0.800	9.87	6.50	0.05	0.129

	Social quality of life				Environment-related quality of life			
Corrected R^2^	0.09				0.21			
	B	SE	β	p	B	SE	β	p
Intercept	60.62	6.41	0	**<0.001**	57.81	5.92	0	**<0.001**
Age	0.15	0.06	0.10	**0.009**	0.24	0.04	0.16	**<0.001**
**Gender**								
Women	Reference category							
Men	-3.54	1.19	-0.08	**0.003**	0.63	0.79	0.14	0.422
**Education status (CASMIN)**								
Low	Reference category							
Medium	6.89	2.91	0.19	**0.018**	7.88	2.09	0.21	**<0.001**
High	7.59	2.76	0.17	**0.006**	11.68	1.98	0.26	**<0.001**
**Employment status**								
Regular working hours	Reference category							
No regular work activity due to COVID-19 restrictions	-3.69	1.95	-0.06	0.059	-3.85	1.24	-0.06	**0.002**
Quarantine (infected with COVID 19 or suspected case)	5.60	5.42	0.02	0.302	-0.70	5.21	-0.00	0.895
Sick leave (illness other than COVID-19)	-0.70	4.23	-0.01	0.868	-6.33	3.11	-0.07	**0.042**
Jobseeker	-4.67	3.31	-0.05	0.158	-5.73	3.06	-0.06	0.061
Housewife/-husband/Pensioner	1.90	2.69	0.03	0.480	0.19	2.04	0.00	0.927
**Place of work**								
Regular	Reference category							
Working from home	-1.18	1.51	-0.02	0.435	-0.25	0.97	-0.01	0.799
Combination working from home/regular	0.67	1.65	0.02	0.685	1.83	1.04	0.04	0.078
Not working regularly	-0.92	2.58	-0.02	0.721	-2.83	2.00	-0.05	0.157
**Profession in the health sector**								
No	Reference category							
Yes	3.44	1.62	0.07	**0.034**	1.29	1.05	0.02	0.217
**Housing situation**								
Access to balcony/garden/terrace	Reference category							
No access to balcony/garden/terrace	0.61	1.47	0.01	0.677	-3.92	0.99	-0.07	**<0.001**
Number of household members	1.45	0.51	0.12	**0.004**	0.45	0.36	0.04	0.205
**Children**								
No	Reference category							
Yes	-4.41	1.48	-0.11	**0.003**	-0.80	1.02	-0.02	0.432
**Combination childcare/home office**								
No	Reference category							
Yes	-2.40	2.68	-0.05	0.370	0.26	1.67	0.00	0.877
**Control variables**								
**Lifetime diagnosis of mental illness**								
No	Reference category							
Yes	-9.28	1.28	-0.23	**<0.001**	-5.61	0.87	-0.14	**<0.001**
**Chronic disease**								
No	Reference category							
Yes	-4.92	1.20	-0.11	**<0.001**	-4.38	0.79	-0.10	**<0.001**
**COVID-19-status**								
Currently suffering from COVID-19	Reference category							
Currently not ill with COVID-19/recovered	-6.43	4.81	-0.04	0.181	1.88	5.37	0.01	0.727

CASMIN=Comparative Analyses of Social Mobility in Industrial Nations, COVID-19=Coronavirus Disease 2019, B=unstandardised beta coefficients, SE=(robust) standard errors, β=standardised beta coefficients, Bold=significant difference (p<0.05)

## References

[1] Schröder C Entringer T Goebel J (2020) Erwerbstätige sind vor dem COVID-19-Virus nicht alle gleich. SOEPpaper (1080). https://www.diw.de/documents/publikationen/73/diw_01.c.789529.de/diw_sp1080.pdf (As at 03.09.2021)

[2] Statistisches Bundesamt (2021) Sonderauswertung zu Sterbefallzahlen der Jahre 2020/2021. https://www.destatis.de/DE/Themen/Gesellschaft-Umwelt/Bevoelkerung/Sterbefaelle-Lebenserwartung/sterbefallzahlen.html (As at 25.08.2021)

[3] DIVI-Intensivregister (2021) Anzahl gemeldeter intensivmedizinisch behandelter COVID-19-Fälle. https://www.intensivregister.de/#/aktuelle-lage/zeitreihen (As at 25.08.2021)

[4] Wachtler B Michalski N Nowossadeck E (2020) Sozioökonomische Ungleichheit und COVID-19 – Eine Übersicht über den internationalen Forschungsstand. Journal of Health Monitoring 5(S7):3–18. https://edoc.rki.de/handle/176904/6965 (As at 03.09.2021)

[5] The WHOQOL Group (1995) The World Health Organization quality of life assessment (WHOQOL): Position paper from the World Health Organization. Soc Sci Med 41(10):1403–1409 8560308 10.1016/0277-9536(95)00112-k

[6] Conrad I Matschinger H Kilian R (2016) WHOQOL-OLD und WHOQOL-BREF – Handbuch für die deutschsprachigen Versionen der WHO-Instrumente zur Erfassung der Lebensqualität im Alter. Hogrefe, Göttingen

[7] Bundeszentrale für gesundheitliche Aufklärung (2020) Gesundheitsbezogene Lebensqualität. https://leitbegriffe.bzga.de/alphabetisches-verzeichnis/gesundheitsbezogene-lebensqualitaet/ (As at 25.08.2021)

[8] Müller KU Samtleben C Schmieder J (2020) Corona-Krise erschwert Vereinbarkeit von Beruf und Familie vor allem für Mütter – Erwerbstätige Eltern sollten entlastet werden. DIW Wochenbericht 87(19):331–340

[9] Robillard R Saad M Edwards J (2020) Social, financial and psychological stress during an emerging pandemic: observations from a population survey in the acute phase of COVID-19. BMJ Open 10(12):e043805 10.1136/bmjopen-2020-043805 PMC7735085 33310814

[10] Hertwig R Liebig S Lindenberger U (2020) Wie gefährlich ist COVID-19? Die subjektive Risikoeinschätzung einer lebensbedrohlichen COVID-19-Erkrankung im Frühjahr und Frühsommer 2020 in Deutschland. SOEPpaper (1095). https://www.diw.de/de/diw_01.c.795737.de/publikationen/soep-papers/2020_1095/wie_gefaehrlich_ist_covid-19__die_subjektive_risikoeinschaet___-erkrankung_im_fruehjahr_und_fruehsommer_2020_in_deutschland.html (As at 03.09.2021)

[11] Di Corrado D Magnano P Muzii B (2020) Effects of social distancing on psychological state and physical activity routines during the COVID-19 pandemic. Sport Sci Health 16(4):619–624 10.1007/s11332-020-00697-5 PMC7517049 32994822

[12] Epifanio MS Andrei F Mancini G (2021) The Impact of COVID-19 Pandemic and Lockdown Measures on Quality of Life among Italian General Population. J Clin Med 10(2):289 10.3390/jcm10020289 PMC7830623 33466778

[13] Pieh C Budimir S Probst T (2020) The effect of age, gender, income, work, and physical activity on mental health during coronavirus disease (COVID-19) lockdown in Austria. J Psychosom Res 136:110186 32682159 10.1016/j.jpsychores.2020.110186 PMC7832650

[14] Teotônio I Hecht M Castro LC (2020) Repercussion of COVID-19 Pandemic on Brazilians’ Quality of Life: A Nationwide Cross-Sectional Study. Int J Environ Res Public Health 17(22):8554 10.3390/ijerph17228554 PMC7698925 33218087

[15] Limbers CA McCollum C Greenwood E (2020) Physical activity moderates the association between parenting stress and quality of life in working mothers during the COVID-19 pandemic. Ment Health and Phys Act 19:100358 10.1016/j.mhpa.2020.100358 PMC7548083 33072187

[16] Qiu J Shen B Zhao M (2020) A nationwide survey of psychological distress among Chinese people in the COVID-19 epidemic: implications and policy recommendations. Gen Psychiatr 33(2):e100213 32215365 10.1136/gpsych-2020-100213 PMC7061893

[17] Suryavanshi N Kadam A Dhumal G (2020) Mental health and quality of life among healthcare professionals during the COVID-19 pandemic in India. Brain Behav 10(11):e01837 32918403 10.1002/brb3.1837 PMC7667343

[18] Huang JZ Han MF Luo TD (2020) Mental health survey of medical staff in a tertiary infectious disease hospital for COVID-19. Chinese Journal of Industrial Hygiene and Occupational Diseases 38(3):192–195 32131151 10.3760/cma.j.cn121094-20200219-00063

[19] Zhang L Ji R Ji Y (2021) Relationship Between Acute Stress Responses and Quality of Life in Chinese Health Care Workers During the COVID-19 Outbreak. Front Psychol 12:599136 33815198 10.3389/fpsyg.2021.599136 PMC8010677

[20] Zinn S Bayer M Entringer T (2020) Subjektive Belastung der Eltern durch Schulschließungen zu Zeiten des Coronabedingten Lockdowns. SOEP paper (1097). https://www.diw.de/documents/publikationen/73/diw_01.c.794185.de/diw_sp1097.pdf (As at 03.09.2021)

[21] Clair R Gordon M Kroon M (2021) The effects of social isolation on well-being and life satisfaction during pandemic. Humanit Soc Sci Commun 8(1):28

[22] Lehberger M Kleih AK Sparke K (2021) Self-reported well-being and the importance of green spaces – A comparison of garden owners and non-garden owners in times of COVID-19. Landsc Urban Plan 212:104108 10.1016/j.landurbplan.2021.104108 PMC9757896 36569995

[23] Kley S Dovbishchuk T (2021) How a Lack of Green in the Residential Environment Lowers the Life Satisfaction of City Dwellers and Increases Their Willingness to Relocate. Sustainability 13(7):3984

[24] Beierle FSJ Vogel K Allgaier.(submitted) CoronaHealth – A Study- and Sensor-based Mobile Technical Framework Exploring Aspects of the COVID-19 Pandemic

[25] Schilling J Buda S Fischer M (2021) Retrospektive Phaseneinteilung der COVID-19-Pandemie in Deutschland bis Februar 2021. Epid Bull 2021(15):8–17

[26] The WHOQOL Group (1996) WHOQOL – Introduction, Administration, Scoring and Generic Version of the Assessment. https://www.who.int/mental_health/media/en/76.pdf (As at 03.09.2021)

[27] König W Lüttinger P Müller WA (1988) A Comparative Analysis of the Development and Structure of Educational Systems. Methodological Foundations and the Construction of a Comparative Educational Scale. CASMIN Working Paper No 12. Universität Mannheim, Mannheim

[28] Team R (2020) RStudio: Integrated Development for R. RStudio, Inc, Boston, MA

[29] Yentes RD Wilhelm F (2018) careless: Procedures for computing indices of careless responding. R package version 1.1.3.

[30] Jacobi F Höfler M Siegert J (2014) Psychische Störungen in der Allgemeinbevölkerung: Studie zur Gesundheit Erwachsener in Deutschland und ihr Zusatzmodul Psychische Gesundheit (DEGS 1-MH). Der Nervenarzt 85:77–87 24441882 10.1007/s00115-013-3961-y

[31] Bidzan-Bluma I Bidzan M Jurek P (2020) A Polish and German Population Study of Quality of Life, Well-Being, and Life Satisfaction in Older Adults During the COVID-19 Pandemic. Front Psychiatry 11:585813 33281646 10.3389/fpsyt.2020.585813 PMC7705096

[32] Herrera MS Elgueta R Fernández MB (2021) A longitudinal study monitoring the quality of life in a national cohort of older adults in Chile before and during the COVID-19 outbreak. BMC Geriatrics 21(1):143 33637054 10.1186/s12877-021-02110-3 PMC7908522

[33] Tenzer F (2020) Anteil der Smartphone-Nutzer in Deutschland nach Altersgruppe im Jahr 2019. https://de.statista.com/statistik/daten/studie/459963/umfrage/anteil-der-smartphone-nutzer-in-deutschland-nach-altersgruppe/ (As at 30.10.2020)

[34] Robert Koch-Institut (2020) Prävention und Management von COVID-19 in Alten- und Pflegeeinrichtungen und Einrichtungen für Menschen mit Beeinträchtigungen und Behinderungen. https://www.rki.de/DE/Content/InfAZ/N/Neuartiges_Coronavirus/Alten_Pflegeeinrichtung_Empfehlung.pdf?__blob=publicationFile (As at 25.08.2021)

[35] Ellert U Kurth BM (2013) Gesundheitsbezogene Lebensqualität bei Erwachsenen in Deutschland. Bundesgesundheitsbl 56(5):643–649 10.1007/s00103-013-1700-y 23703481

[36] Ikeda T Igarashi A Odani S (2021) Health-Related Quality of Life during COVID-19 Pandemic: Assessing Impacts of Job Loss and Financial Support Programs in Japan. Appl Res Qual Life:1–17 10.1007/s11482-021-09918-6 PMC7846494 33552309

[37] Reisch T Heiler G Hurt J.(eingereicht) Behavioral gender differences are reinforced during the COVID-19 crisis. https://arxiv.org/abs/2010.10470 (As at 03.09.2021) 10.1038/s41598-021-97394-1 PMC8478918 34584107

[38] Cohrdes C Pryss R Baumeister.(eingereicht) “Help, I Need Somebody…” – Seeking Support as One Key Coping Strategy for Maintaining Quality of Life during the COVID-19 Pandemic

[39] Morfeld M Bullinger M Nantke J (2005) Die Version 2.0 des SF-36 Health Survey – Ergebnisse einer bevölkerungsrepräsentativen Studie. Soz Praventivmed 50(5):292–300 16300173 10.1007/s00038-005-4090-6

[40] Mielck A Vogelmann M Schweikert B (2010) Gesundheitszustand bei Erwachsenen in Deutschland: Ergebnisse einer repräsentativen Befragung mit dem EuroQol 5D (EQ-5D). Das Gesundheitswesen 72:476–486 19802780 10.1055/s-0029-1239508

[41] Lipskaya-Velikovsky L (2021) COVID-19 Isolation in Healthy Population in Israel: Challenges in Daily Life, Mental Health, Resilience, and Quality of Life. Int J Environ Res Public Health 18(3):999 33498662 10.3390/ijerph18030999 PMC7908389

[42] De Witte H (1999) Job Insecurity and Psychological Well-being: Review of the Literature and Exploration of Some Unresolved Issues. Eur J Work Organ Psychol 8:155–177

[43] McKee-Ryan F Song Z Wanberg C (2005) Psychological and Physical Well-Being During Unemployment: A Meta-Analytic Study. J Appl Psychol 90:53–76 15641890 10.1037/0021-9010.90.1.53

[44] Winkelmann L Winkelmann R (1998) Why Are the Unemployed So Unhappy? Evidence from Panel Data. Economica 65(257):1–15

[45] Qi M Li P Moyle W (2020) Physical Activity, Health-Related Quality of Life, and Stress among the Chinese Adult Population during the COVID-19 Pandemic. Int J Environ Res Public Health 17(18):6494 10.3390/ijerph17186494 PMC7558071 32906604

[46] Abel T Bruhin E Sommerhalder K (2018) Health Literacy/Gesundheitskompetenz. https://www.leitbegriffe.bzga.de/alphabetisches-verzeichnis/health-literacy-gesundheitskompetenz/ (As at 25.08.2021)

[47] KKH – Kaufmännische Krankenkasse (2020) Plötzlich Vollzeitpapa: Kinder größter Stressfaktor in der Krise. https://www.kkh.de/presse/pressemeldungen/ploetzlich-vollzeitpapa-nachwuchs-jetzt-groesster-stressfaktor (As at 14.09.2021)

[48] Wang X Lei SM Le S (2020) Bidirectional Influence of the COVID-19 Pandemic Lockdowns on Health Behaviors and Quality of Life among Chinese Adults. Int J Environ Res Public Health 17(15):5575 10.3390/ijerph17155575 PMC7432516 32748825

[49] Groarke JM Berry E Graham-Wisener L (2020) Loneliness in the UK during the COVID-19 pandemic: Cross-sectional results from the COVID-19 Psychological Wellbeing Study. PLOS ONE 15(9):e0239698 32970764 10.1371/journal.pone.0239698 PMC7513993

[50] Umberson D Pudrovska T Reczek C (2010) Parenthood, Childlessness, and Well-Being: A Life Course Perspective. J Marriage Fam 72(3):612–629 21869847 10.1111/j.1741-3737.2010.00721.x PMC3159916

[51] Huebener M Spieß C Siegel N (2020) Wohlbefinden von Familien in Zeiten von Corona: Eltern mit jungen Kindern am stärksten beeinträchtigt. DIW Wochenbericht 30/31:527–537

[52] Möhring K Naumann E Reifenscheid M (2021) The COVID-19 pandemic and subjective well-being: longitudinal evidence on satisfaction with work and family. Eur Soc 23(sup1):S601–S617

[53] World Health Organization (1998) Programme on mental health: WHOQOL User Manual. World Health Organization, Geneva

[54] Skevington SM Lotfy M O’Connell KA (2004) The World Health Organization’s WHOQOL-BREF quality of life assessment: psychometric properties and results of the international field trial. A report from the WHOQOL group. Qual Life Res 13(2):299–310 15085902 10.1023/B:QURE.0000018486.91360.00

[55] Brähler E Mühlan H Albani C (2007) Teststatistische Prüfung und Normierung der deutschen Versionen des EUROHIS-QOL Lebensqualität-Index und des WHO-5 Wohlbefindens-Index. Diagnostica 53(2):83–96

[56] Zurek M Friedmann L Kempter E (2021) Haushaltsklima, Alleinleben und gesundheitsbezogene Lebensqualität während des COVID-19-Lockdowns in Deutschland. Präv Gesundheitsf: 1–8 10.1007/s11553-021-00865-6 PMC8231074 40477268

[57] Lingelbach K Janssen D Maurer P (2021) Gesellschaftliche und psychologische Auswirkungen der COVID-19-Pandemie in Deutschland – Ergebnisse einer Online-Befragung. https://publica.fraunhofer.de/eprints/urn_nbn_de_0011-n-6305986.pdf (As at 03.09.2021)

